# Initial Cardiac Rhythm Correlated to Emergency Department Survival

**DOI:** 10.4137/cmc.s695

**Published:** 2009-02-09

**Authors:** Rade B. Vukmir

**Affiliations:** Critical Care Medicine Associates, Sewicley, PA 15143, U.S.A. Department of Emergency Medicine, University of Pittsburgh Medical Center, Pittsburgh, PA 15213, U.S.A.

**Keywords:** cardiac rhythm, cardiac arrest, prehospital, emergency, arrhythmia

## Abstract

**Background:**

This study attempted to correlate the initial cardiac rhythm and survival from prehospital cardiac arrest, as a secondary end-point.

**Methods:**

Prospective, randomized, double-blinded clinical intervention trial where bicarbonate was administered to 874 prehospital cardiopulmonary arrest patients in prehospital urban, suburban, and rural emergency medical service environments.

**Results:**

This group’s manifested an overall survival rate of 13.9% (110 of 793) of prehospital cardiac arrest patients. The most common presenting arrhythmia was ventricular fibrillation (VF) (45.0%), asystole (ASY) (34.4%), and pulseless electrical activity (PEA) (15.7%). Less commonly found were normal sinus rhythm (NSR) (1.8%), other (1.8%), ventricular tachycardia (VT) (0.6%), and atrioventricular block (AVB) (0.5%) as prearrest rhythms.

The best survival was noted in those with a presenting rhythm of AVB (57.1%), VT (33.3%), VF (15.7%), NSR (14.3%), PEA (11.2%), and ASY (11.1%) (p = 0.02). However, there was no correlation between the final cardiac rhythm and outcome, other than an obvious end-of-life rhythm.

**Conclusion:**

The most common presenting arrhythmia was VF (45%), while survival is greatest in those presenting with AVB (57.1%).

## Introduction

The US emergency medicine services (EMS) experience was reported by Crampton in 1975 noting a 26% decline in prehospital and 62% in-hospital mortality involving those who underwent ambulance transport, who were <70 years of age, noting a 66% success rate in prehospital cardiopulmonary resuscitation (CPR) measured as long-term survival under optimal circumstances.^1^

Iseri reported experience with 26 primary cardiac arrest patients and rapid response paramedic units demonstrating optimal resuscitation in the ventricular fibrillation (VF) group (14) which was amenable to successful countershock therapy in 86% (12) resulting in survival in 43% (6).^2^ They also defined a poor outcome cohort, the brady-systolic cardiac arrest group, which was associated with autopsy proven coronary artery disease in 50% (7) of patients and was found to be universally fatal. Interestingly, they concluded that a more aggressive approach to prehospital management of brady-systolic arrests was warranted.

Eisenburg reported the results of an evaluation of prehospital care by Emergency Medical Technicians (EMTs) compared to that delivered after the addition of paramedic skills, such as defibrillation, endotracheal intubation and drug administration to the resuscitation armaterium.^3^ They reported an improved rate of survival to the coronary care unit (19% to 34%) and rate of hospital discharge (7% to 17%), which they related to a decrease in time delay to advanced care delivery, which was decreased to one-third from 27.5 to 7.7 minutes.

The use of prehospital health care providers to intervene in acute cardiac emergencies has historically been a focus of emergency care. However, Dean reported on the outcome of 134 patients who received mobile paramedic unit care compared to control patients without paramedic intervention demonstrating no change in outcome by multiple logistic regression analysis.^4^ Defibrillation was the only beneficial intervention identified, but also added a 29 minute delay to hospital arrival suggesting the need for more streamlined care.

Later, Shuster went on to evaluate 15 prehospital studies during the early years of emergency medical care suggesting no benefit of prehospital administration of any of a number of commonly administered prehospital medications.^5^

Four factors are related to the ability to resuscitate patients in prehospital arrest: time of starting rescue procedures, use of electrical defibrillation, accuracy of technique of basic life support (BLS), and ventilation efficacy decreasing in utility.^6^

The “early defibrillation” controversy has once again raised interest in utilization of first responders or EMT in a two-tier response system. Wilson evaluated 126 patients whose care was limited to BLS: mask oxygen, intravenous (IV) fluids, closed chest massage, and artificial respiration.^7^ The survival rate was 22% (28) to hospital admission and 9% (11) to hospital discharge, with a favorable prognosis group identified to include those with initial rhythm of VF or tachycardia, 14% (7 of 50); and initial blood pressure > 90 mmHg and pulse rate >50 bpm, 50% (3 of 6). However, if the patient was in cardiac arrest then CPR did not change outcome, other than an obvious end-of-life rhythm.

Our study attempted to correlate the outcome in prehospital cardiac arrest to the initial cardiac rhythm documented pre-arrest, as a secondary endpoint.

## Methods

This prospective, randomized, multicenter clinical trial involved cardiac arrest patients encountered by advanced paramedics (EMT-P) in a prehospital setting, and transported to hospitals usually within the study area, usually a 5–30 minute transport radius.

Inclusion criteria were subjects suffering from cardiac arrest refractory to defibrillation in whom intravenous access was obtained. Patients received standard advanced cardiac life support (ACLS) protocol including chest compressions, ventilation, defibrillation, epinephrine (0.01 mg/kg), atropine (0.01 mg/kg), and antiarrhythmics or pressor agents as warranted. Patients were individually randomized to a treatment group receiving empiric dose of sodium bicarbonate (Abbott, Chicago, U.S.A) 1 ample (50 mEq/L) early in the arrest cycle. The control group received an equal amount of normal saline in a double-blinded fashion to clarify the benefits of the osmolar load versus base deficit correction. Routine demographic and clinical variables related to outcome were analyzed including demographics, response to bicarbonate administration, scene factors, response time, cardiopulmonary variables, procedures, and duration of arrest (Table 1).

Routine cardiopulmonary variables were monitored. Specifically, presentation and final cardiac rhythm such as VF, asystole (ASY), pulseless electrical activity (PEA), normal sinus rhythm (NSR), ventricular tachycardia (VT), and atrioventricular block (AVB) were noted. Patient outcome was recorded as the return of spontaneous circulation (ROSC) and initial emergency department survival as a primary endpoint.

Patients were enrolled under the Doctrine of Implied Consent for the emergency use of an accepted resuscitation modality and notification was provided if requested by family or healthcare resources. Their hospital records were not reviewed.

In addition, administration of an FDA approved agent (sodium bicarbonate) in the emergency setting for moderate to prolonged arrest may be the standard of care, and in conjunction with the above conditions which are met, consent could be waived. This study, was approved by the University of Pittsburgh Institutional Review Board, using this rationale.

Therefore, sodium bicarbonate administered in the emergency setting to a cardiac arrest patient classifies as minimal risk intervention. Thus, the criteria that were fulfilled by this study included minimal risk, no adverse influence on rights as such emergency research could not be practicably carried out, while subjects and/or their families would be provided with additional information at their request.

Numerical data was represented as means and standard deviation with Student’s t-test, Fisher’s exact, Chi Square with Pearson correlation tests utilized for logistic regression intergroup comparison (alpha < 0.05) (SPSS/PC+^®^, Chicago, IL). The study results were examined by the investigators at three month intervals (or 25% of projected patients) to verify early trends and outcome with capability of later modification.

The sample size of 1,000 was sufficient to delineate a 50% difference in survival and neurologic outcome at 80% power and a 95% confidence interval between control and treatment groups. This estimate was based on a 12% rate of ROSC. This was a single step emergent intervention excluding the need for stopping rules.

## Results

**Figure 1.**
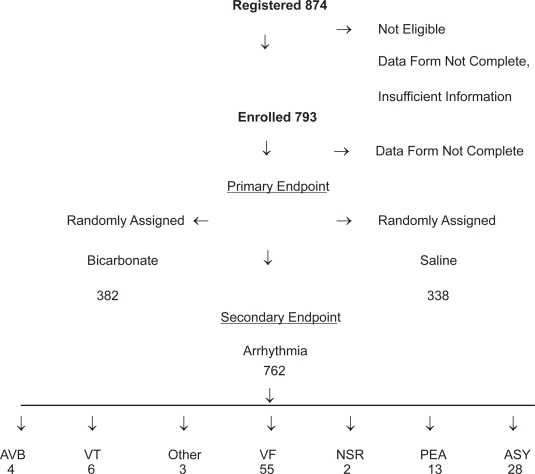
Trial Profile.

The overall survival rate was 13.9% (110 of 793) of prehospital cardiac arrest patients. (Fig. 1)

The most commonly encountered cardiac rhythm was VF (45.0%), ASY (15.7%), NSR (1.8%), other (1.8%), VT (0.6%), and AVB (0.5%) (Table 2).

However, optimal survival was noted in those patients presenting with AVB (57.1%), VT (33.3%), VF (15.7%), NSR (14.3%), PEA (11.2%), and ASY (11.1%) (p = 0.02) (Tables 3 and 4).

There was no correlation between the final cardiac rhythm and outcome, other than an obvious end-of-life rhythm.

## Discussion

Prehospital predictors of outcome may potentially be inferred by the analysis of animal experimental data. Angelos evaluated a ten minute VF and five minute BLS resuscitation model to identify improved coronary perfusion in the normal neurologic outcome group as an independent predictor of favorable outcome.^8^ The author has performed a similar trial in brief (five minute), moderate (ten minute), and prolonged (15 minute) canine VF model to also identify improved coronary perfusion pressure (CPP) and systemic mean arterial pressure as favorable outcome predictors associated with improved survival and neurologic outcome.^9^

Paradis in a study where the CPP quantified as the aortic to right atrial pressure gradual during the relaxation phase, correlated to the ROSC.^10^ In those patients with ROSC, the initial CPP was increased (13.4 vs. 1.6 mmHg), as was the maximal CPP obtained (25.6 vs. 8.4 mmHg). They found that only those with a CPP > 15 mmHg had ROSC, although not all 75% (18 of 24) of those with adequate coronary perfusion were successfully resuscitated.

Brison’s demographic analysis of the cardiac resuscitation experience of 1510 cardiac arrest patients where 92.1% of patients were 50 years of age, 68.3% were male, and 79.6% of arrests occurred at home.^11^ The average ambulance response time of witnessed events was 7.8 minutes with an overall survival rate of 2.5%. Factors predicting survival include: age, ambulance response time, whether CPR was started before ambulance arrival, but interestingly was not related to early defibrillation.

Tresch evaluated a population of 381 cardiac arrest patients comparing older and younger (<20 years) cohorts, who have undergone paramedic witnessed cardiac arrest.^12^ The elderly patient cohort more commonly had a past history of heart failure (25 vs. 10%) was more commonly taking digoxin (40 vs. 20%), diuretics (35 vs. 25%), and were more likely to complain of dyspnea (53 vs. 40%). Younger patients were more likely to complain of chest pain (27 vs. 13%) and presented in VF (42 vs. 22%). Interestingly, the patients’ chief complaint correlated with initial rhythm where 68% of those with chest pain demonstrated a VF event compared to 21% of those with dyspnea. Although, there were equivalent initial resuscitation rates in the elderly their survival to discharge was decreased comparatively (24 to 10%).

Survey data offered by Ng concerning 105 younger arrests (1–39 years) patients found a male predominance (62%), was secondary to cardiac disease (38%) due to atherosclerotic heart disease in 50% and secondary toxic exposure in 21%.^13^ The most common presenting rhythm was VF (45%) associated with a 48% resuscitation rate with over 28% of post-resuscitation patients progressing to long-term survival. Favorable outcome was predicted by the arrest being witnessed, or associated with primary cardiac dysrhythmia; while asystole was a negative prognostic indicator. Age, sex, race, bystander CPR, and paramedic response time were not significant prognostic factors affecting long-term survival.

The effect of an extended EMS training program on cardiac arrest survival was evaluated in 1196 patients by Wright, where the majority of patients 62% (740) presenting in electromechanical dissociation (EMD) or ASY, while 38% (456) presented in VF.^14^ The survival rate in those who presented with asystole was dismal 0.1% (1 of 740). Factors associated with the likelihood of presenting in ventricular fibrillation include age < 71 years, witnessed arrest, bystander resuscitation, public arrest, and ambulance response time <6 minutes. While improved outcome was associated with shorter response time, but not bystander CPR with the newly acquired skills used in 78% of patients.

Clearly, there are widely discrepant rates of survival in hospital compared to prehospital cardiac arrest events. Rosenberg evaluated 300 hospitalized patients demonstrating a 54% initial post-CPR survival followed by 23% survival to hospital discharge.^15^ Predictors of good resuscitation outcome include an initial ventricular tachycardia or fibrillation rhythm, and brief duration of CPR < 30 minutes.

Prehospital survival was suboptimal compared to in-hospital events due to inherent logistic considerations. Roth reported on 187 cases of out of hospital arrest where an improved outcome was noted based on initial rhythm—VF/VT (15.3%) compared to other rhythms-including ASY, idioventricular (IVR), AVB, and EMD (3.4%), as well as with bystander CPR improving survival to 24% in VF/VT and 0% in other rhythms.^16^

Secondly, response times of less than four minutes resulted in improved survival to discharge in 23.1 compared to 7.0% of VF/VT events, and 30.8 compared to 7.7% of other arrhythmic events (13). Likewise, the use of bystander CPR improved outcome from 23.1 to 42.9% in VF/VT and 7.7 to 15.8% when ACLS providers arrived within four minutes.^14^

The overall survival rate of 13.9% (110 of 793 of our study) patients compares favorably to a 3.8% (1.7%–13%) pooled analysis of 3220 prehospital arrest patients suggesting improved prehospital outcome in this study.^17^

The predominant proportion (80%) of patients presenting in prehospital cardiac arrest are found to have VF (45%) or asystole (35%). Certainly, it is appropriate to direct the major emphasis of care toward the VF population, suggested to have the best chance at achieving ROSC based on the rapid defibrillation intervention.

However, the probability of successful resuscitation was more likely with AVB (57.1%) and VT (33.3%) than for VF (15.7%).

It is better to define the AVB-bradycardia group as lower risk with its own attendant etiology usually associated with to less significant cardiac pathophysiology. However, we are then left with the likelihood of Type II error, to failing to conclude a difference between groups when in fact limited by small sample size.

Focusing on the ventricular tachycardia-fibrillation continuum associated with more significant cardiac pathophysiology we find adequate sample size—80% of the total population to reach conclusions concerning likelihood of survival in this group.

Therefore, it seems that initial cardiac rhythm serves as only a possible predictor of outcome with organized rhythms AVB vs. PEA/ASY associated with a higher resuscitation rate than less organized rhythms VT vs. VF in this present content.

## Figures and Tables

**Table 1.** Prehospital variables correlated to survival.

**Demographic**
Age, Weight, Gender
**Response Time**
ET Arrest, ET By CPR, ET BLS, ET ACLS, ET ROSC, ET Hosp
**Interventions**
Bicarbonate (Dose, Weight based)
**Scene Factor**
Bystander CPR, Witnessed
**Cardiopulmonary Variables**
Initial Rhythm, Initial Systolic Blood Pressure (ISBP), IDBP
**Procedures**
Intubation, IV, Other
**Duration of Arrest**
Short (<5 min), Moderate (5–15 min), Long-term (>15 min)
**EMS Coverage**
Urban, Suburban, Rural
**Past Medical History**
MI, HTN, DM, CHF, COPD, CABG
**Medication**
Cardiac, HTN, Arrhythmia, Pulmonary, Hematologic, GI, Psychiatric, Seizure

**Table 2.** Incidence of initial cardiac rhythm.

**Rhythm**	**Incidence (%)**	**Patients (n = 76%)**
VF	45.0	295
ASY	34.4	225
PEA	15.7	103
SINUS	1.8	12
Other	1.8	12
VT	0.6	4
AVB	0.5	3
N	0.2	1

**Table 3.** Survivorship related to initial cardiac rhythm.

**Rhythm**	**Survival (%)**	**Patients (n = 762)**
AVB	57.1	4
VT	23.3	6
Other	20.0	3
VF	15.9	55
NSR	14.3	2
PEA	11.2	13
ASY	11.1	28

**Table 4.** Initial rhythm correlated to ER survival.

**IRHYTHM**	**ER Survival**		**Patients**
	**No**	**Yes**	
No	1		1
	100.0		0.1
	0.2		
VF	295	55	350
	84.3	**15.7**	45.9
	45.0	51.4	
VT	4	2	
	66.7	**33.3**	6
	0.6	1.9	0.8
ASY	225	28	253
	88.9	**11.1**	33.2
	34.4	26.2	
PEA	103	13	116
	88.8	**11.2**	15.2
	15.7	12.1	
AVB	3	4	7
	42.9	**57.1**	0.9
	0.5	3.7	
Sinus	12	2	14
	85.7	**14.3**	1.8
	1.8	1.9	
Other	12	3	15
	80.0	20.0	2.0
	**1.8**	**2.8**	
	655	107	762
	86.0	14.0	100.0

Chi Square with Pearson correlation (p = 0.2).
